# The Effect of Cardiovascular Medications on Disease-Related Outcomes in Idiopathic Pulmonary Fibrosis: A Systematic Review and Meta-Analysis

**DOI:** 10.3389/fphar.2021.771804

**Published:** 2021-11-11

**Authors:** Wan-Tong Zhang, Xu-Jie Wang, Chun-Miao Xue, Xin-Yu Ji, Lin Pan, Wei-Liang Weng, Qiu-Yan Li, Guo-Dong Hua, Bao-Chen Zhu

**Affiliations:** ^1^ Xiyuan Hospital, China Academy of Chinese Medical Sciences, Beijing, China; ^2^ Dongzhimen Hospital, Beijing University of Chinese Medicine, Beijing, China; ^3^ Institute of Basic Research in Clinical Medicine, China Academy of Chinese Medical Sciences, Beijing, China; ^4^ National Clinical Research Center for Chinese Medicine Cardiology, Beijing, China

**Keywords:** idiopathic pulmonary fibrosis, ACEI/ARB, statin, anticoagulants, meta-analysis

## Abstract

**Background:** Multiple studies have revealed that idiopathic pulmonary fibrosis (IPF) patients are more at risk for cardiovascular diseases and that many IPF patients receive cardiovascular medications like statins, angiotensin-converting enzyme inhibitor (ACEI), angiotensin receptor blocker (ARB), and anticoagulants. Existing studies have reported divergent findings on the link between cardiovascular medications and fibrotic disease processes. The aim of this study is to synthesize the evidence on the efficacy of cardiovascular medications in IPF.

**Methods:** We searched studies reporting the effect of cardiovascular medications on IPF in the PubMed, Embase, Web of Science, Cochrane Library, and two Chinese databases (China National Knowledge Infrastructure database and China Wanfang database). We calculated survival data, forced vital capacity (FVC) decline, and IPF-related mortality to assess the efficacy of cardiovascular medications in IPF. We also estimated statistical heterogeneity by using I^2^ and Cochran Q tests, and publication bias was evaluated by risk of bias tools ROBINS-I.

**Results:** A total of 12 studies were included in the analysis. The included studies had moderate-to-serious risk of bias. Statin use was associated with a reduction in mortality (hazard ratio (HR), 0.89; 95% CI 0.83–0.97). Meta-analysis did not demonstrate any significant relationship between statin use and the FVC decline (HR, 0.86; 95% CI 0.73–1.02), ACEI/ARB use, and survival data (HR, 0.92; 95% CI 0.73–1.15) as well as anticoagulant use and survival data (HR, 1.16; 95% CI 0.62–2.19).

**Conclusion:** Our study suggested that there is a consistent relationship between statin therapy and survival data in IPF population. However, there is currently insufficient evidence to conclude the effect of ACEI, ARB, and anticoagulant therapy on IPF population especially to the disease-related outcomes in IPF.

## Introduction

Idiopathic pulmonary fibrosis (IPF) is a specific form of interstitial lung disease (ILD) (Raghu et al., 2011), characterized by progressive decline in lung function, worsening dyspnea, and impaired health-related quality of life. It generally progresses relentlessly and carries the poorest prognosis of the chronic idiopathic interstitial pneumonias, with a median survival of 3–5 years (Bjoraker et al., 1998). The pathogenesis of IPF is poorly understood. It is hypothesized that alveolar epithelial cell injury triggers release of cytokines such as transforming growth factor beta 1 (TGF-β1), platelet-derived growth factor (PDGF), and tumor necrosis factor (TNF) α. Cardiovascular diseases (Hyldgaard et al., 2014) are common comorbidities in patients with IPF, and many patients with IPF are receiving medications to reduce cardiovascular risk such as statins, anti-hypotentive agents, and anticoagulants. Statins are well known for its lipid-lowering properties through inhibition of 3-hydroxy-3-methylglutaryl coenzyme-A (HMG-CoA) reductase (LaRosa et al., 1999). *In vitro* study on lung fibroblasts showed that simvastatin can inhibit the induction of collagen production by TGF-β (Oka et al., 2013). *In vivo* study showed that simvastatin treatment severely reduced fibrosis, bronchiole adventitial collagen, and bronchiole epithelium (Kruzliak et al., 2015). Angiotensin-converting enzyme inhibitors (ACEIs) and angiotensin receptor blockers (ARBs) are widely used in the management of cardiovascular diseases and systemic hypertension (Chobanian et al., 2003). ACEI inhibits angiotensin I from converting to angiotensin II by inhibiting the activity of angiotensin converting enzyme, thus promoting vasodilation through the kallikrein–kinin system (KKS) to achieve the purpose of lowering blood pressure (Turner and Kodali, 2020). ARB directly inhibits the binding of angiotensin II type 1 receptor (AT1) with angiotensin II receptor (Piepho, 2000). In an *in vitro* study, ACEI has potential anti-pulmonary fibrosis effect by inhibiting apoptosis of human lung epithelial cells (Uhal et al., 1998). An *in vivo* study has proven that ACEI can alleviate focal alveolar lesions, alleviate lung inflammation and pulmonary fibrosis, and improve the survival rate of pulmonary fibrosis rats (Gao et al., 2013; Molteni et al., 2007). Anticoagulants are commonly used to combat thrombotic disorders such as atrial fibrillation and strokes. Recent studies have shown a systemic prothrombotic state in people with IPF (Navaratnam et al., 2014). Several researches with large datasets from different populations reveal an increased risk of vascular events in IPF patients (Hubbard et al., 2008; Sode et al., 2010; Sprunger et al., 2012; Schulman and Crowther, 2012). Also, coagulant and plasminergic proteases are likely to have fibrotic actions, which involve cell-mediated responses via receptors (e.g., PARs and uPAR) and coreceptors (e.g., integrins) (Schuliga, et al., 2018). Based on the above studies, it is thought that there may be a potential benefit to the use of anticoagulants in patients with IPF. There is increasing awareness that the broader pharmacologic properties of statins, ACEI, ARB, and anticoagulants encompass the abilities to modulate local fibroproliferative pathways in a variety of organ systems or prolong the survival of IPF patients.

Existing studies have reported divergent findings on the link between medications used for cardiovascular diseases and fibrotic disease processes. The potential effect of these medications in interstitial fibrosis remains unclear. IPF remains a progressive disease, and new antifibrotic therapies do not reverse existing fibrosis. To address this knowledge gap, we conducted a systematic review and meta-analysis of all medications used in cardiovascular diseases to evaluate their relative efficacy and safety for IPF. Therefore, evaluating the effect of the potential adjuvant therapy may offer new approaches in the management of IPF.

## Methods

Our systematic review and meta-analysis was conducted under the Cochrane Handbook (Higgins et al., 2020). Reporting was consistent with the Preferred Reporting Items for Systematic Reviews and Meta-Analyses (PRISMA) (Guise et al., 2017).

### Search Strategy

We systematically searched PubMed, Embase, Web of Science, Cochrane Library (Cochrane Central Register of Controlled Trials), and two Chinese databases (China National Knowledge Infrastructure database and China Wanfang database) to identify non-randomized studies of cardiovascular medications for IPF published from January 2000 to July 2021, with no language restriction. The full search strategy was employed using combinations of MeSH terms and text words around “statin,” “hydroxymethylglutaryl-CoA reductase inhibitors,” “ACEI,” “angiotensin-converting enzyme inhibitors,” “angiotensin converting enzyme inhibitors,” “ARB,” “angiotensin receptor antagonists,” “angiotensin receptor blockers,” “anticoagulants,” “anticoagulant drugs,” “idiopathic pulmonary fibrosis,” “IPF,” “pulmonary fibroses,” “non-randomised studies,” “retrospective studies,” “prospective studies,” “cohort studies,” “case-control studies,” and “clinical trial.” Additional studies were derived from screening the reference lists of included non-randomized studies and previous systematic reviews.

### Eligibility Criteria

We included the studies with the following criteria: 1) they included participants with diagnosis of IPF; 2) intervention involving any form of statins, ACEI, ARB, and anticoagulants; 3) controls including placebo or other IPF therapy; and 4) reporting disease-related outcomes including mortality and pulmonary function.

### Data Extraction

Two independent authors (W-TZ and B-CZ) assessed all studies for eligibility, extracted the data, and assessed study quality. Disagreements were resolved by consensus-based discussion. The full-text articles were downloaded, and the same inclusion criteria were used to decide whether to include or exclude articles. Each reviewer carried out the quality assessment of each selected article, assessed the completeness of the data extraction, and confirmed the quality rating to reduce bias independently.

### Quality Assessment

Three authors (W-TZ, B-CZ, and X-JW) evaluated all the included studies. Any disagreement was solved through discussion and rechecking of the article. For the included studies, the Cochrane risk of bias tool ROBINS-I was used to assess the risk of bias (Sterne et al., 2016), including 1) confounding, 2) selection of participants into the study, 3) classification of interventions, 4) intended intervention, 5) missing data, 6) measurement of outcomes, and 7) selection of the reported results and other possible sources of bias. In this way, all articles selected for inclusion in the review were graded under the categories of low, moderate, serious, critical, or no information risk of bias.

### Data Synthesis

Primary outcome measurements of survival data were performed. Since survival data include survival outcomes such as survival rate, mortality, censoring, and survival time, we merged the survival data in the meta-analysis. Secondary outcomes were forced vital capacity (FVC) decline and IPF-related mortality. Where more than one study reported the same outcome measured in similar way, a meta-analysis was undertaken. Alternatively, a narrative synthesis of the findings was conducted. For meta-analysis, we calculated a hazard ratio (HR) and 95% CIs using a fixed-effects model. Heterogeneity of included studies was quantified using the chi-squared test (Q test) and I^2^ statistic. We used RevMan software for statistical analysis.

## Results

### Study Selection and Characteristics of the Included Studies

**FIGURE 1:**
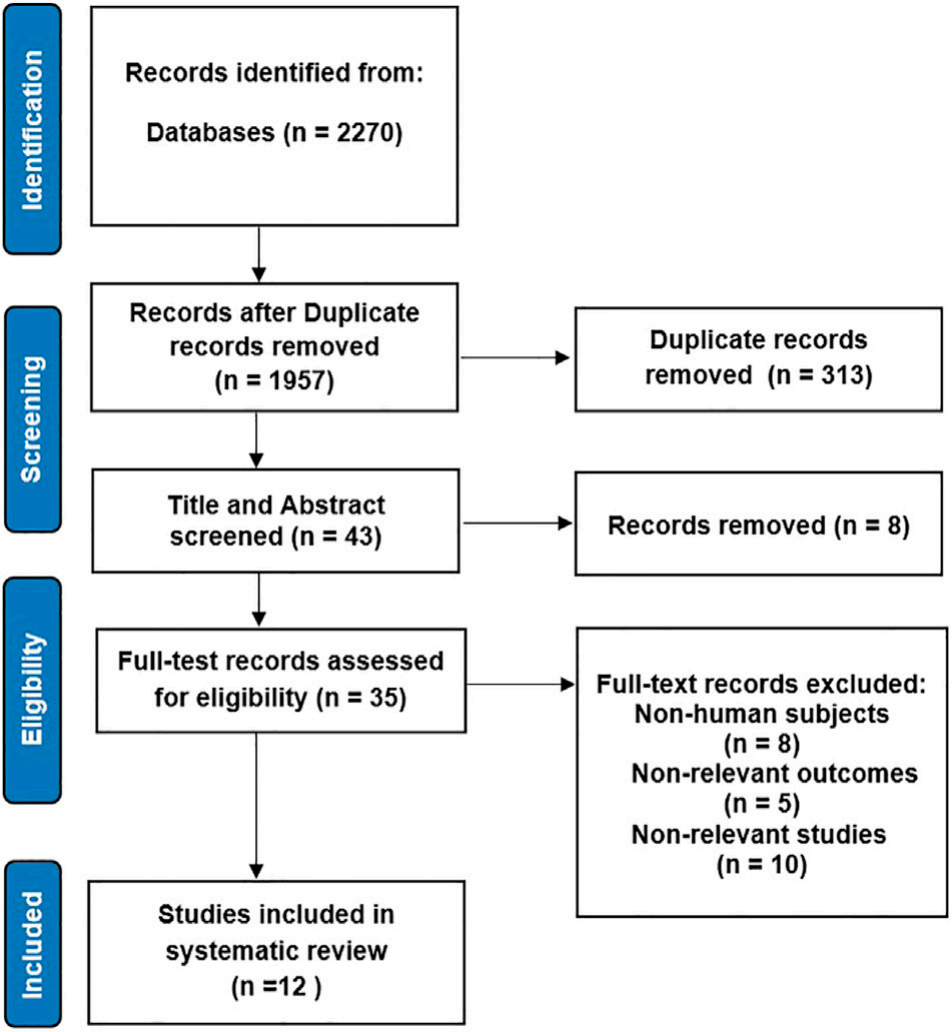
Flowchart.

We initially identified 2,270 potentially relevant publications through database searches and other sources. After deleting 313 duplicate articles, we screened the titles and abstracts of 43 related articles. Thirty-five articles were selected for full-text review. Ultimately, 12 studies met our inclusion criteria and were included for the final analysis (Figure 1). The characteristics of studies included in the systematic review and meta-analysis are shown in Table 1.

**TABLE 1:** Characteristics of the included studies.

Type	Author, year	Country	Study design	Intervention	Control	Outcome	Conclusion
Statin	Nadrous et al. (2004)	United States	Retrospective	Statin *n* = 35	No statin	Mean survival HR 0.97; 95% CI 0.62–1.52 (*p* = 0.985)	These data do not suggest a beneficial effect of statins on survival in patients with IPF.
					*N* = 442		
	Vedel-Krogh et al. (2015)	Denmark	Retrospective	Statin *n* = 261	No statin	All-cause Mortality HR 0.76; 95% CI 0.62–0.93 (*p* = 0.05)	Among patients with interstitial lung disease, statin use was associated with reduced all-cause mortality.
					*N* = 522		
	Ekström and Bornefalk-Hermansson (2016)	Sweden	Prospective cohort study	Statin *n* = 122	No statin	All-cause mortality HR 1.13; 95% CI 0.81–1.57	There was no association between statin treatment and survival.
					*N* = 340		
	Kreuter et al. (2017)	United States	Prospective population-based study	Statin *n* = 276	No statin	All-cause mortality HR 0.54; 95% CI 0.24–1.21 (*p* = 0.1369)	This study indicated that statins may have a beneficial effect on clinical outcomes in IPF.
					*N* = 348	IPF-related mortality HR 0.36; 95% CI 0.14–0.95 (*p* = 0.0393)	
					—	FVC decline or death HR 0.71; 95% CI 0.48–1.07 (*p* = 0.1032)	
—	Kreuter et al. (2018)	INPULSIS	Retrospective	Statin *n* = 312	No statin	Annual rate of decline in FVC mean	It showed a numerically lower FVC decline in placebo-treated patients who were receiving statins at baseline.
					*N* = 749	Difference 50.8 ml/year; 95% CI 10.9–112.5 (*p* = 0.1065)	
					—	FVC decline or death HR 1.01; 95% CI 0.72–1.41 (*p* = 0.9700)	
—	Durheim et al. (2020)	United States	Retrospective cohort study	Statin *n* = 4,775	No statin	In-hospital mortality OR 0.76; 95% CI 0.67–0.87 (*p* < 0.001)	Statins significantly associated with a lower risk of in-hospital mortality.
					*N* = 4,892		
—	Lambert et al. (2021)	Belgium	Retrospective	Statin *n* = 171	No statin	Survival HR 0.82; 95% CI 0.50–1.35 (*p* = 0.428)	This study demonstrated a significant beneficial effect of statin therapy on IPF evolution, particularly on FVC and DLCO decline, but not significantly affecting survival.
					*N* = 152	Annual FVC% difference 2.9%, CI 1.6–4.4 (*p* < 0.001)	
					—	DLCO% decline difference 1.3%, CI 0.24–2.3 (*p* = 0.013)	
Anticoagulants	Kubo et al. (2005)	Japan	Prospective study	Warfarin *n* = 23	No warfarin *n* = 33	Survival HR 2.9; 95% CI 1.0–8.0 (*p* = 0.04)	Anticoagulant therapy has a beneficial effect on survival in patients with IPF.
—	Tomassetti et al. (2013)	Italy	Retrospective cohort study	Warfarin *n* = 25	None *n* = 25	Survival HR 4.8; 95% CI 1.8–12.8 (*p* = 0.002)	In this retrospective study, patients treated with anticoagulants had a worse survival and a shorter interval to disease progression.
						Interval to disease progression HR 2.7; 95% CI 1.2–6.5 (*p* = 0.023)	
—	Kreuter et al. (2017)	United States	Prospective study	Anticoagulants *n* = 32	No anticoagulants *n* = 592	All-cause mortality HR 2.6; 95% CI 0.7–8.8 (*p* = 0.136)	This post-hoc analysis suggests that anticoagulants used for non-IPF indications may have unfavorable effects in IPF patients
						IPF-related mortality HR 4.7; 95% CI 1.1–19.3 (*p* = 0.034)	
—	King et al. (2021)	United States	Retrospective cohort study	Direct oral anticoagulants (DOACs) *n* = 57	No anticoagulants *n* = 1,070	All-cause mortality or transplant HR 1.368; 95% CI 0.500–3.737	In patients with IPF, DOACs were not associated with increase in risk of death or transplant and reduction in transplant-free survival.
						Transplant-free survival HR 1.510; 95% CI 0.709–3.215	
—	King et al. (2021)	United States	Retrospective cohort study	Warfarin *n* = 49	No anticoagulants *n* = 1,070	All-cause mortality or transplant HR 2.566; 95% CI 1.095–6.0165	In patients with IPF, warfarin was associated with increase in risk of death or transplant and reduction in transplant-free survival.
						Transplant-free survival HR 2.101; 95% CI 1.025–4.307	
—	Lambert et al. (2021)	Belgium	Retrospective review	Anticoagulants *n* = 49	No anticoagulants 274	Survival HR 1.25; 95% CI 0.71–2.21 (*p* = 0.444)	This study suggests that anticoagulant use showed a trend towards worse DLCO decline in IPF patients, but no effect on survival.
						DLCO decline (difference −1.3%, CI 2.6–0.02 (*p* = 0.055)	
ACEI/ARB	Nadrous et al. (2004)	United States	Retrospective	ACEI *n* = 52	No ACEI/ARB *N* = 425	Mean survival HR 1.05; 95% CI 0.73–1.52 (*p* = 0.776)	These data do not suggest a beneficial effect of ACEI on survival in patients with IPF.
—	Ekström and Bornefalk-Hermansson (2016)	Sweden	Perspective cohort study	ACEI/ARB *n* = 161	No ACEI/ARB *N* = 301	All-cause mortality HR 0.63; 95% CI 0.47–0.85	ACEI/ARB treatment is related to improving the survival of IPF patients.
—	Kreuter et al. (2019)	United States	Prospective population-based study	ACEI *n* = 111	No ACEI/ARB *N* = 392	All-cause mortality HR 1.2; 95% CI 0.5–2.8 (*p* = 0.737)	This analysis suggests that ACEI treatment may potentially be associated with slightly better outcomes in patients with IPF.
						Disease progression HR 0.6; 95% CI 0.4–0.9 (*p* = 0.026)	
—	Kreuter et al. (2019)	United States	Prospective population-based study	ARB *n* = 121	No ACEI/ARB *N* = 392	All-cause mortality HR 2.2; 95% CI 1.2–4.3 (*p* = 0.017)	ARB treatment may potentially be associated with greater risk of all-cause.

Note. The INPULSIS studies were performed at 205 sites in 24 countries in the Americas, Europe, Asia, and Australia.

HR, hazard ratio; IPF, idiopathic pulmonary fibrosis; FVC, forced vital capacity; DLCO, diffusing capacity of the lungs for carbon monoxide; DOACs, direct oral anticoagulants; ACEI, angiotensin-converting enzyme inhibitor; ARB, angiotensin receptor blocker.

### Risk of Bias

**FIGURE 2:**
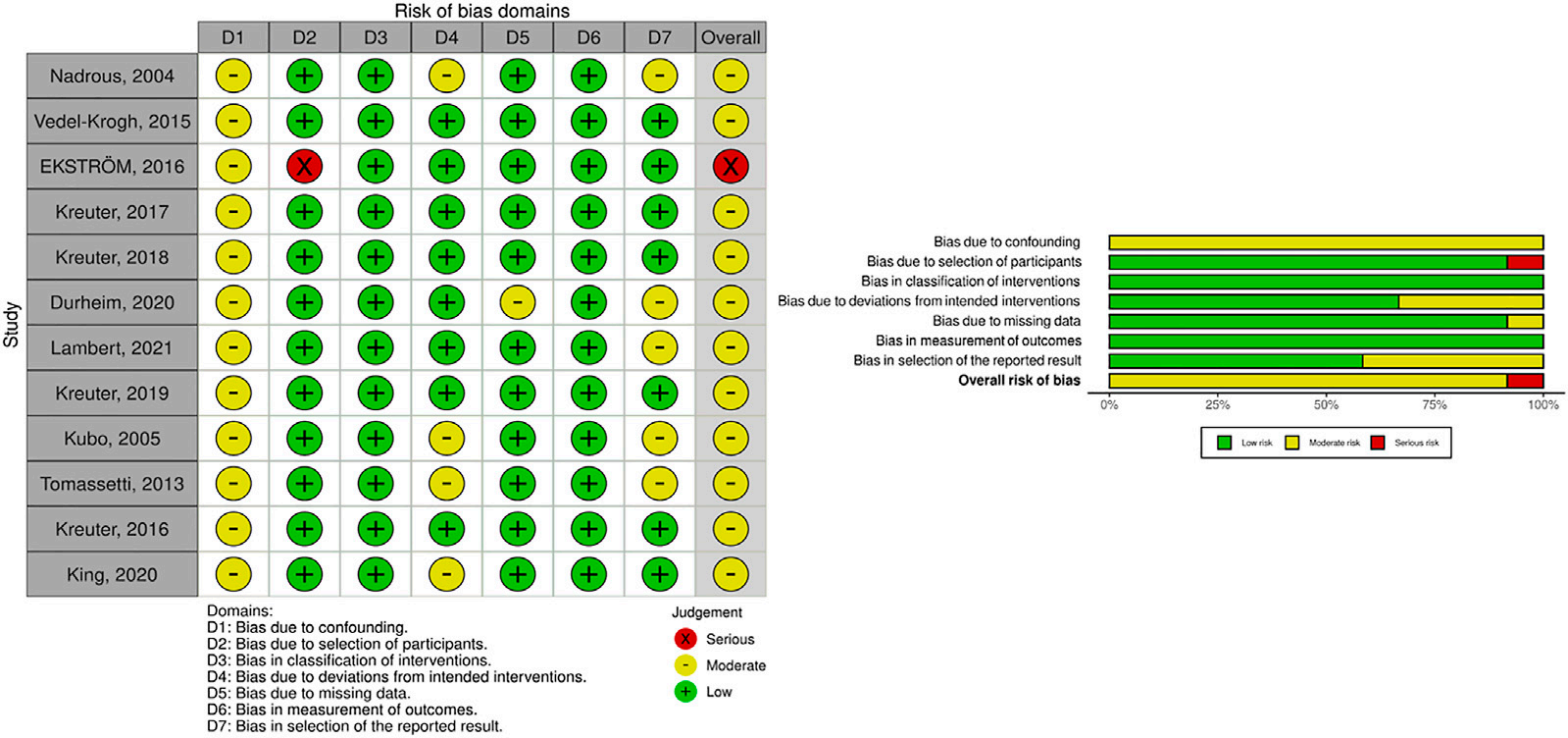
Risk of bias graph.

A total of 12 non-randomized studies were included in this study, of which seven were retrospective studies and five were prospective studies. The above studies were reasonable for non-randomized studies, but none of them could achieve the quality of well-implemented randomized trials. Therefore, the overall bias was assessed as moderate-to-serious risk (Figure 2). The lack of diagnostic criteria for IPF in a prospective cohort study (Ekström and Bornefalk-Hermansson, 2016) based on the National Swedevox Register, where approximately 80% of the patients had IPF, assessed the bias in selection of participants as high risk. One retrospective cohort study (Durheim Michael T, 2020) had data from 740 hospitals in the United States, but the data registration information was not stated in the article, so the bias due to missing data was assessed as moderate risk. Although five studies (Nadrous Hassan F, 2004; Durheim Michael T, 2020; M Lambert Eline, 2021; Kubo et al., 2005; Tomassetti S, 2013) had no indications for selective reporting, they lacked evidence support such as prior registration information or data analysis plan, so their bias in selection of the reported result was assessed as a moderate risk of bias.

### Meta-Analysis Result of Statin

**FIGURE 3:**
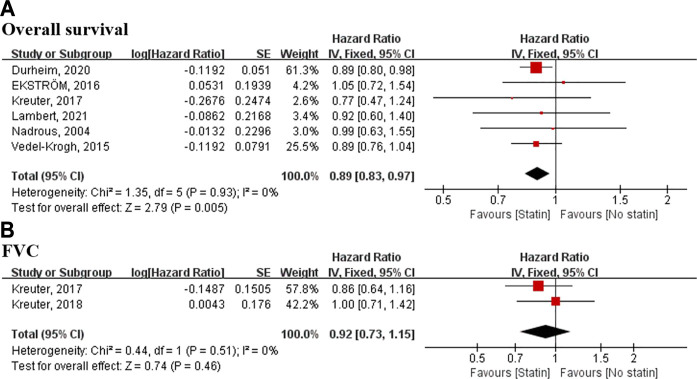
Meta-analyses of the included studies examining overall survival and FVC for statin vs. no statin. The marker size indicates the relative weight of the study, as it contributes to the results of the overall comparison. IV indicates inverse-variance random-effects analysis; HR, hazard ratio; FVC, forced vital capacity.

Of seven included statin studies, six studies reported the survival data. In the fixed-effects meta-analysis, statin use was associated with a reduction in mortality (HR, 0.89; 95% CI 0.83–0.97) (Figure 3A).

Two studies involving 1,685 patients reported on a composite measure of FVC decline of >10% or death. The meta-analysis did not demonstrate any significant relationship between statin use and the composite outcome (HR, 0.86; 95% CI 0.73–1.02) (Figure 3B).

### Meta-Analysis Result of Angiotensin-Converting Enzyme Inhibitor/Angiotensin Receptor Blocker

**FIGURE 4:**
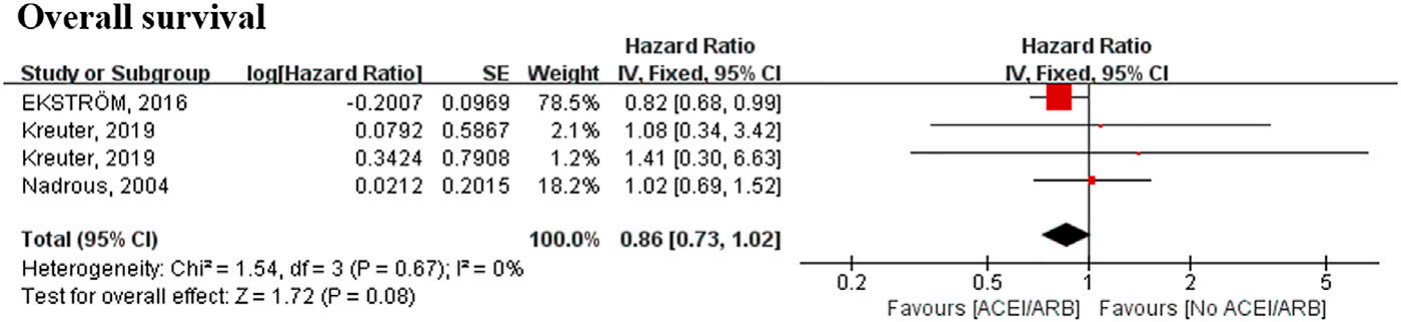
Meta-analyses of the included studies examining overall survival for ACEI/ARB vs. no ACEI/ARB. The marker size indicates the relative weight of the study, as it contributes to the results of the overall comparison. IV indicates inverse-variance random-effects analysis; HR, hazard ratio; ACEI, angiotensin-converting enzyme inhibitor; ARB, angiotensin receptor blocker.

Four studies involving 1,955 patients reported a relationship between ACEI/ARB use and survival data. The meta-analysis did not demonstrate any significant relationship between ACEI/ARB use and survival data (HR, 0.92; 95% CI 0.73–1.15) (Figure 4).

### Meta-Analysis Result of Anticoagulants

**FIGURE 5:**
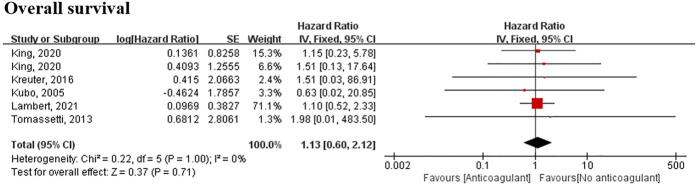
Meta-analyses of the included studies examining overall survival for anticoagulant vs. no anticoagulant. The marker size indicates the relative weight of the study, as it contributes to the results of the overall comparison. IV indicates inverse-variance random-effects analysis; HR, hazard ratio.

Six studies involving 2,172 patients reported a relationship between anticoagulant use and survival data. Although the meta-analysis did not demonstrate any significant relationship between anticoagulant use and survival data (HR, 1.16; 95% CI 0.62–2.19) (Figure 5), five of six studies included in the meta-analysis showed that patients given anticoagulant medication remained at increased risk of mortality.

## Discussion

IPF is defined as a chronic, progressive, fibrosing ILD of unknown etiology. Multiple studies have revealed associated pulmonary as well as extra-pulmonary comorbidities (Buendía-Rold´an et al., 2017). Likewise, patients are more at risk for cardiovascular disorders such as ischemic heart disease, atrial fibrillation, and thrombotic vascular events. To reduce their cardiovascular risk, many IPF patients receive cardiovascular medications like statins, ACEI, ARB, and anticoagulants (Hubbard, et al., 2008). Existing studies have reported divergent findings on the link between medications used for cardiovascular diseases and fibrotic disease processes. Given the increasing awareness that pharmacologic properties of statins, ACEI, ARB, and anticoagulants encompass the abilities to modulate fibroproliferative pathways, therefore, identifying the treatment with the best safety and efficacy is important. This systematic review and meta-analysis including 12 non-randomized studies, covering four types of cardiovascular medications, aimed to evaluate the relationship between cardiovascular medications and IPF. To the best of our knowledge, this is currently the first systematic review and meta-analysis investigating different types of cardiovascular medications for IPF. Our study suggested a consistent relationship between statin therapy and all-cause mortality in IPF population. There was no statistically significant association between ACEI/ARB use and decline in FVC. Meanwhile, although the meta-analysis did not demonstrate any significant relationship between anticoagulant use and survival data, we found that all the six studies related to survival data showed the result that patients given anticoagulant medication remained at increased risk of mortality.

Regarding the statin use in IPF patients, of seven included studies, five studies reported survival analysis, through which three studies suggested a beneficial effect of statins on survival. One study reported IPF-related mortality, which suggested that statin use at baseline significantly reduced chance of IPF-related death. Two studies reported on a composite measure of FVC decline of >10% or death with no statistically difference. Regarding the ACEI/ARB use in IPF patients, of four included studies, only one study suggested that ACEI/ARB treatment is related to improving the survival of IPF patients, same with the result of meta-analysis. Regarding the anticoagulant use in IPF patients, of six included studies, only one study suggested that warfarin has a beneficial effect on survival in patients with IPF. Interestingly, others all indicated that patients treated with anticoagulants had a worse survival. Above all, we found that among the cardiovascular medications, only statin was beneficial to survival data of IPF patients. However, since only one study reported IPF-related death, we could not specify the true pharmacological action on IPF clearly. More studies focused on the outcome of IPF-related death should be conducted in the future.

This study has some limitations. Firstly, we concentrated our research on published literatures and did not screen gray literature. This is because the use of the risk of bias tools ROBINS-I requires very explicit and comprehensive reporting of the primary research study. Secondly, most studies used all-cause mortality and survival rate but not IPF-related death as their primary outcomes. So the causes of death were missing in the majority of studies. Thirdly, all studies included are non-randomized, and half of the studies are retrospective. Therefore, there exists some bias due to missing data and confounding that cannot be avoided. Given these challenges, larger-scale samples and higher-quality studies are needed in the future to draw conclusions about the exact causal relationship between survival data of IPF and cardiovascular medications.

Nevertheless, a crucial strength of the present study lies in the large number of included patients and full analysis of the relationship between different types of cardiovascular medications and survival data. Lastly, we would like to propose two suggestions for future clinical trials especially randomized controlled trials (RCTs) of cardiovascular medications for IPF. The combination, especially with antifibrotic drugs, could maximize the treatment effects of cardiovascular medications, but the majority of included trials were not clarified. If the included patients are grouped according to different antifibrotic treatments or non-antifibrotic treatments, then we can then classify and synthesize the outcome data as different types of syndromes in the systematic review, and the analyzed results can provide more pointed guidance for clinical practice. On the other hand, the quality of life of patients was not reported in most of the included studies. As IPF is defined as a chronic, progressive ILD, the multidimensional quality of life is an indispensable indicator and evaluation tool to show the trend of treatment effectiveness. We suggested that future prospective trials should be improved in related fields.

## Conclusion

Our study suggested a consistent relationship between statin therapy and survival data in IPF population. However, there is currently insufficient evidence to conclude the effect of ACEI, ARB, and anticoagulant therapy on IPF population especially to the disease-related outcomes in IPF. Considering the limitations of available literature, we would recommend a prospective RCT or cohort study that captures IPF-related outcomes of cardiovascular medications and use of concurrent antifibrotic treatment.
